# Identification of potential prognostic small nucleolar RNA biomarkers for predicting overall survival in patients with sarcoma

**DOI:** 10.1002/cam4.3361

**Published:** 2020-08-11

**Authors:** Jianwei Liu, Xiwen Liao, Xianze Zhu, Peizhen Lv, Rong Li

**Affiliations:** ^1^ Department of Spine Surgery The Third Affiliated Hospital of Guangxi Medical University Nanning People's Republic of China; ^2^ Department of Hepatobiliary Surgery The First Affiliated Hospital of Guangxi Medical University Nanning People's Republic of China; ^3^ Department of Reproductive Center The Third Affiliated Hospital of Guangxi Medical University Nanning People's Republic of China

**Keywords:** overall survival, risk score, sarcoma, small nucleolar RNA, The Cancer Genome Atlas

## Abstract

**Objective:**

The main purpose of the present study is to screen prognostic small nucleolar RNA (snoRNA) markers using the RNA‐sequencing (RNA‐seq) dataset of The Cancer Genome Atlas (TCGA) sarcoma cohort.

**Methods:**

The sarcoma RNA‐seq dataset comes from the TCGA cohort. A total of 257 sarcoma patients were included into the prognostic analysis. Multiple bioinformatics analysis methods for functional annotation of snoRNAs and screening of targeted drugs, including biological network gene ontology tool, Gene Ontology (GO), Kyoto Encyclopedia of Genes and Genomes (KEGG), Gene Set Enrichment Analysis (GSEA), and connectivity map (CMap) are used.

**Results:**

We had identified 15 snoRNAs that were significantly related to the prognosis of sarcoma and constructed a prognostic signature based on four prognostic snoRNA (U3, SNORA73B, SNORD46, and SNORA26) expression values. Functional annotation of these four snoRNAs by their co‐expression genes suggests that some of them were closely related to cell cycle‐related biological processes and tumor‐related signaling pathways, such as Wnt, mitogen‐activated protein kinase, target of rapamycin, and nuclear factor‐kappa B signaling pathway. GSEA of the risk score suggests that high risk score phenotype was significantly enriched in cell cycle‐related biological processes, protein SUMOylation, DNA replication, p53 binding, regulation of DNA repair, and DNA methylation, as well as Myc, Wnt, RB1, E2F, and TEL pathways. Then we also used the CMap online tool to screen five targeted drugs (rilmenidine, pizotifen, amiprilose, quipazine, and cinchonidine) for this risk score model in sarcoma.

**Conclusion:**

Our study have identified 15 snoRNAs that may be serve as novel prognostic biomarkers for sarcoma, and constructed a prognostic signature based on four prognostic snoRNA expression values.

## Graphical abstract

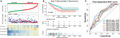

## 1 INTRODUCTION

Malignant tumors that occur in mesoderm are collectively called sarcomas.^1^, ^2^, ^3^ The basic strategy for routine treatment of sarcomas is comprehensive treatment, that is surgical treatment combined with chemotherapy, and chemotherapy has become an important means of clinical treatment of sarcoma.^4^ Sarcoma, like other malignant tumors, has tumor heterogeneity, and the genomic characteristics in tumor tissue often predict the therapeutic response and prognosis of sarcoma. Small nucleolar RNA (snoRNA) is a type of small noncoding RNA that widely exists in the nucleolus of eukaryotic cells. It has a length of 0‐300 nt and has conservative structural elements. SnoRNAs mainly consist of two families: C/D box snoRNAs and H/ACA box snoRNAs.^5^ Its function mainly directs the dioxymethylation and pseudo uracil ization of ribosomal RNA through base pairing. In addition, there are also reports that snoRNA is involved in posttranscriptional modification of snRNA, tRNA, and mRNA.^5^, ^6^ With the development of sequencing technology, more and more data indicate that snoRNA is abnormally regulated and functions in cancers.^6^, ^7^, ^8^, ^9^, ^10^ However, the functional mechanisms and clinical values of snoRNAs in sarcomas have not been reported. The Cancer Genome Atlas (TCGA) database is a tumor database containing more than 30 tumor multi‐omics whole‐genome sequencing datasets, including sarcoma multi‐omics datasets, as well as sarcoma RNA‐seq dataset.^11^, ^12^ Because there are few reports on the clinical application value and functional mechanism of snoRNAs in sarcoma, the main purpose of this study is to screen prognostic snoRNA biomarkers using the RNA‐seq dataset of TCGA sarcoma cohort.

## 2 MATERIALS AND METHODS

### 2.1 Sources of data

The snoRNAs were extracted from the RNA‐seq dataset of TCGA sarcoma cohort (https://portal.gdc.cancer.gov, version 13.0).^12^ The normalization of the RNA‐seq dataset used the *edgeR* package in the R platform.^13^ We obtained a total of 940 snoRNAs. By excluding snoRNAs with an average expression level of less than 1 in the R platform using *edgeR* package, a total of 107 snoRNAs were obtained for subsequent analysis. The clinical indicators of sarcoma were obtained from the University of California, Santa Cruz (UCSC) Xena (http://xena.ucsc.edu). A total of 257 patients were included in the follow‐up survival analysis by including patients with RNA‐seq dataset and clinical indicators in this study.

### 2.2 Prognostic snoRNAs screening and risk score model construction

The prognostic snoRNA screening was performed using the multivariate Cox proportional risk regression model in the R platform using the *survival* package (https://cran.r‐project.org/web/packages/survival/index.html) by adjusting for the clinical parameters of age, residual tumor, and tumor multifocal. Then, the prognostic snoRNAs were screened using the *step* function to construct the optimal risk score model, with a smallest *P* value. The weight coefficients (β) of each snoRNA were generated from the multivariate Cox regression model.^14^, ^15^, ^16^, ^17^ The risk score was calculated as follows: risk score = ExpsnoRNA_1_ × βsnoRNA_1_ + ExpsnoRNA_2_ × βsnoRNA_2_ + … ExpsnoRNA_n_ × βsnoRNA_n_ (Exp: expression level). We then used the Kaplan‐Meier algorithm of *survivalROC* package (https://cran.r‐project.org/web/packages/survivalROC/index.html) in the R platform to evaluate the prognostic accuracy of this risk score model. We also used the risk score model and clinical indicators to construct a nomogram model to assess its contribution to patient prognosis, and developed an individualized prognostic scoring system based on this risk score and traditional clinical indicators. The nomogram was generated by *rms* package (https://cran.r‐project.org/web/packages/rms/index.html) in the R platform. We also developed a joint effect survival analysis to assess the value of risk score in combination with clinical indicators in the clinical outcome prediction of sarcomas.

### 2.3 Function exploration of prognostic snoRNAs in sarcomas

SnoRNAs mainly exist in the intron region of protein‐coding genes or nonprotein‐coding genes, and participate in posttranscriptional modification of snRNA, tRNA, and mRNA. Therefore, we can screen the snoRNA co‐expression encoding genes for functional annotation of specific snoRNAs to understand their biological functions in specific diseases. Co‐expression encoding genes of snoRNAs were screened by the *Cor* function in the R platform using the Pearson correlation coefficient (*r*). Functional enrichment analysis of snoRNA co‐expression encoding genes using Database for Annotation, Visualization, and Integrated Discovery v6.8 (DAVID v6.8, https://david.ncifcrf.gov/home.jsp) online tool,^18^ and Biological Networks Gene Ontology tool (BiNGO).^19^

### 2.4 Functional annotation of the risk score

In order to further understand the influence of this risk score on the prognosis of sarcomas, we used Gene Set Enrichment Analysis (GSEA, https://www.gsea‐msigdb.org/gsea/index.jsp) to annotate the function of this risk score model.^20^ The parameter setting of GSEA software is set according to its default procedure. Results with nominal *P* < .05, |Normalized Enrichment Score (NES)| > 1, and false discovery rate (FDR) < 0.25 were considered statistically significant.^20^ At the same time, we also used the edgeR package to screen differentially expressed genes (DEGs) between patients in the high‐risk and low‐risk groups, so as to further understand the differences in biological functions and pathways between patients with different risks. The criteria for DEG are as follows: |log_2_ fold change (FC)| > 2, *P* < .05, and FDR < 0.05. At the same time, we also used the connectivity map (CMap, https://portals.broadinstitute.org/cmap/) online tool to screen the targeted drugs in sarcoma for this risk score.^21^, ^22^ The chemical structure of these drug was obtained from PubChem (https://pubchem.ncbi.nlm.nih.gov),^23^ and the drug‐gene interaction relationship was obtained from STICH (http://stitch.embl.de),^24^, ^25^, ^26^ which both were online tools.

### 2.5 Statistical analysis

False discovery rate is performed according to Benjamini‐Hochberg procedure.^27^ Log‐rank test is used for Kaplan‐Meier curve analysis. Univariate and multivariate Cox proportional risk regression models were used to assess prognostic differences between groups. Volcano plot and heat map were drawn by *ggplot2*. The R platform version is R3.6.2, and the Cytoscape was version 3.6.1. *P* < .05 believes that the difference reaches statistical significance.

## 3 RESULTS

### 3.1 Survival analysis and risk score model construction

**Figure 1:**
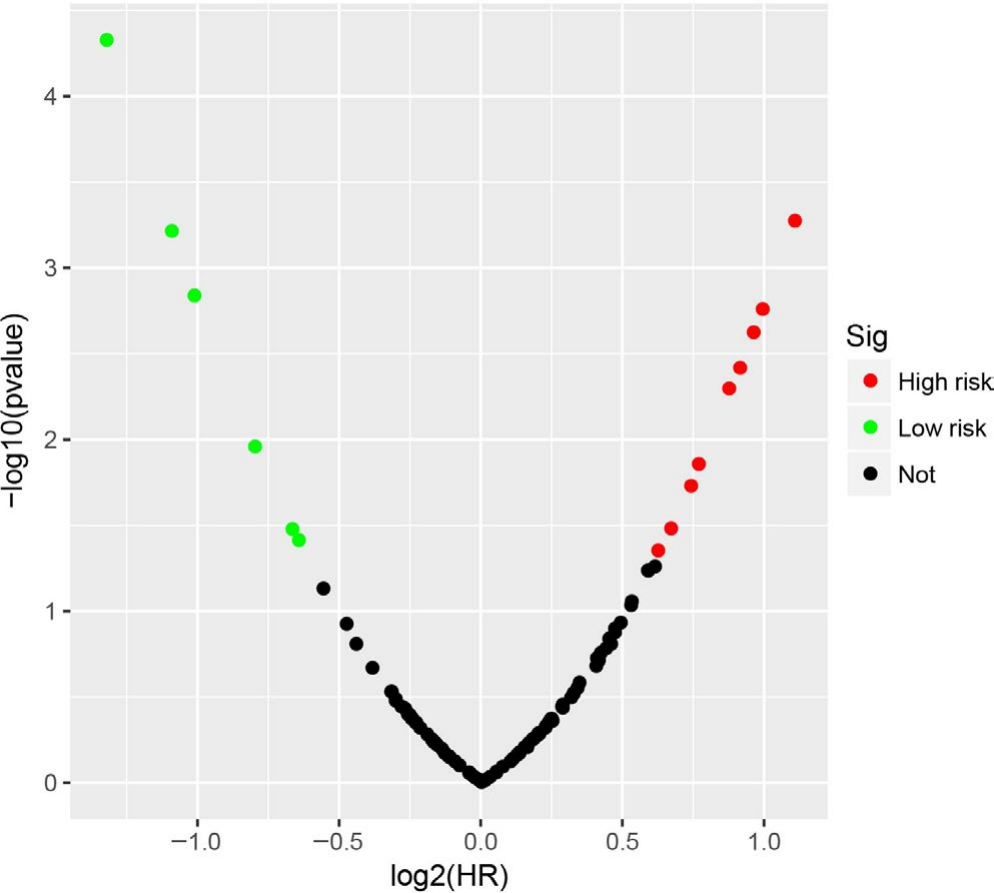
Survival analysis results of snoRNAs in sarcoma

**Figure 2:**
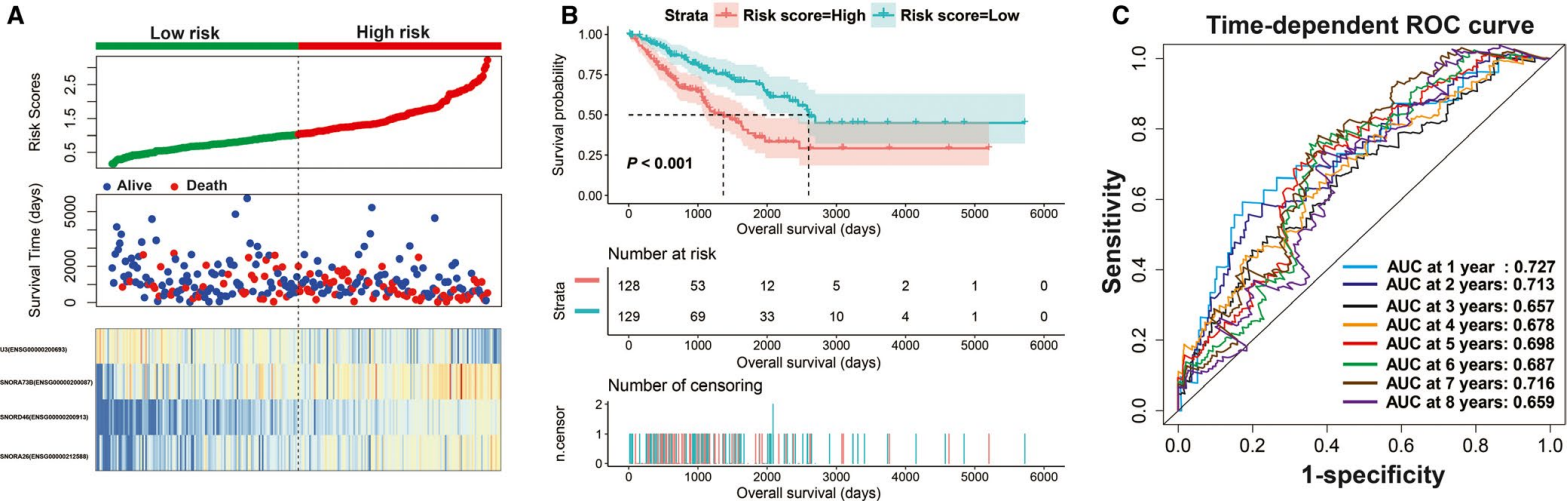
Prognosis evaluation of the risk score model based on four prognostic snoRNAs. A, Distribution map of risk score, survival time, and status; B, Kaplan‐Meier curves of risk score; C, Time‐dependent ROC curve of risk score

**Figure 3:**
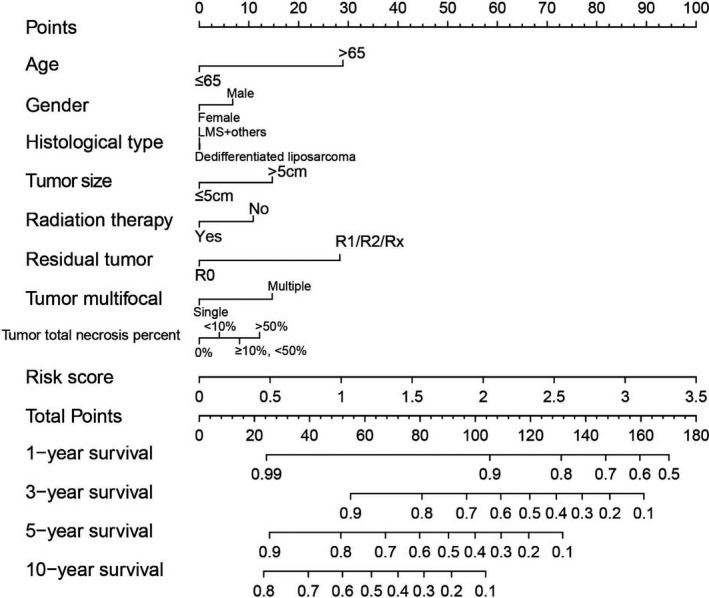
Nomogram of the risk score model in sarcoma

**Figure 4:**
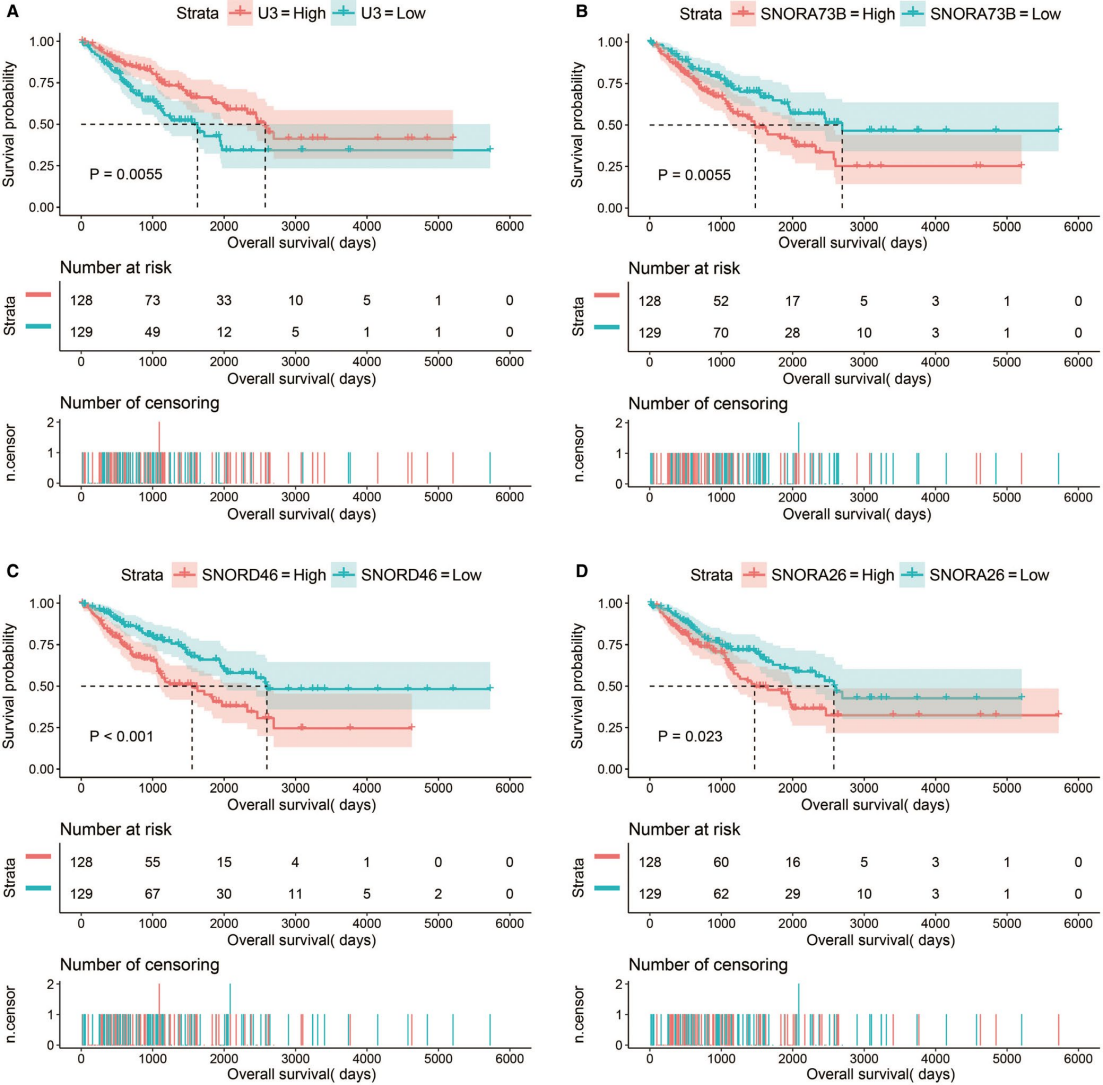
Kaplan‐Meier curves of four prognostic snoRNAs. A, Kaplan‐Meier curves of U3; B, Kaplan‐Meier curves of SNORA73B; C, Kaplan‐Meier curves of SNORD46; D, Kaplan‐Meier curves of SNORA26

**Figure 5:**
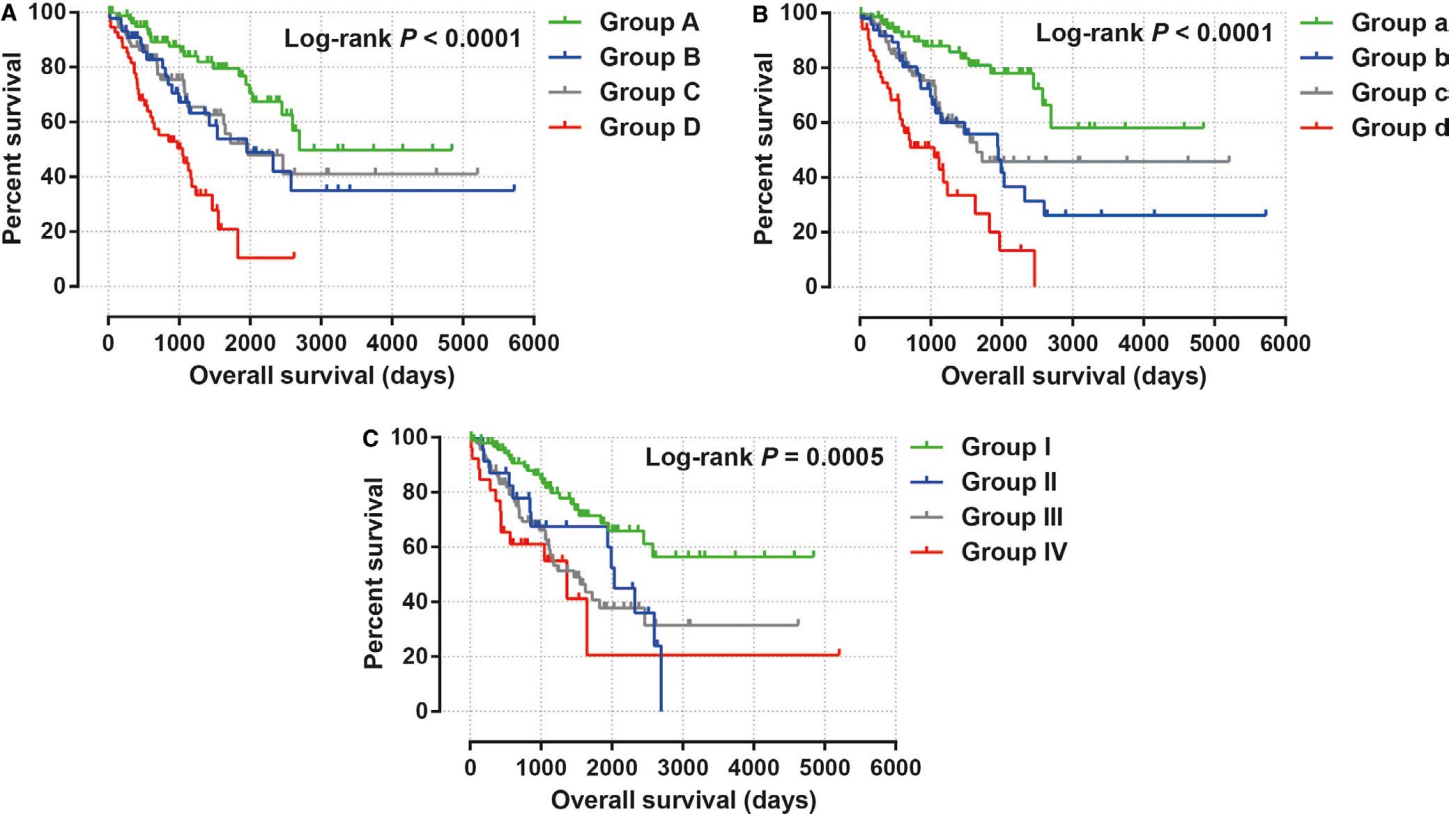
Joint effect survival analysis of the risk score model and clinical indicators in sarcoma. A, Joint effect of age and risk score in sarcoma prognosis; B, Joint effect of residual tumor and risk score in sarcoma prognosis; C, Joint effect of tumor multifocal and risk score in sarcoma prognosis

The demographic data of TCGA sarcoma patients are summarized in Table S1. A total of 257 sarcoma patients were included in the prognosis analysis. We performed a multivariate survival analysis of 107 snoRNAs and found that 15 snoRNAs were significantly correlated with the prognosis of sarcomas in TCGA cohort (Table S2; Figure 1). Then, we had constructed a four‐snoRNAs prognostic signature as a risk score model for sarcomas. The four snoRNAs are: U3 (ENSG00000200693), SNORA73B (ENSG00000200087), SNORD46 (ENSG00000200913), and SNORA26 (ENSG00000212588). The calculation formula of risk score is as follows: risk score = Exp_U3_ × (−0.1803) + Exp_SNORA73B_ × 0.1826 + Exp_SNORD46_ × 0.2846 + Exp_SNORA26_ × 0.1613. Distribution of risk score suggests that patients with high risk have a poor prognosis (log‐rank *P* < .001, adjusted *P* < .001, HR = 2.378, 95% CI = 1.560‐3.625, Figure 2A,B). Time‐dependent receiver operating characteristic (ROC) curve suggests that this risk score is reliable in predicting the prognosis of sarcomas, and the maximum area under the curve is 0.727 for 1‐year prediction (Figure 2C). The nomogram constructed based on this risk score model and clinical parameters indicates that this risk score model contributes the most to the prognosis of sarcomas (score ranked between 0 and 100, Figure 3). The Kaplan‐Meier curves of these four snoRNAs are shown in Figure 4A‐D. We found that patients with low U3 expression were associated with a poor clinical outcome, while high expression of SNORA73B, SNORD46, and SNORA26 was correlated with poor prognosis, respectively. Further analysis showed that the joint effect survival analysis of the risk score model and clinical indicators could markedly distinguish the subgroups of patients with different survival outcomes (Figure 5A‐C; Table 1).

**Table 1:** Joint effect survival analysis of the risk score model and clinical parameters in sarcoma

Group	Risk score	Variables	Patients (n = 257)	MST (d)	Crude HR (95% CI)	Crude *P*	Adjusted HR (95% CI)	Adjusted *P* ^c^
		Age (y)						
A	Low risk	≤65	84	2694	1		1	
B	Low risk	>65	45	1953	2.025 (1.071‐3.830)	.030	1.848 (0.959‐3.558)	.066
C	High risk	≤65	73	1970	1.905 (1.062‐3.417)	.031	2.128 (1.172‐3.861)	.013
D	High risk	>65	55	1049	4.918 (2.784‐8.689)	<.001	4.895 (2.741‐8.742)	<.001
		Residual tumor^a^						
a	Low risk	R0	77	NA	1		1	
b	Low risk	R1/R2/Rx	51	1953	2.939 (1.532‐5.640)	.001	2.297 (1.159‐4.553)	.014
c	High risk	R0	76	1649	2.519 (1.347‐4.712)	.004	2.383 (1.266‐4.485)	.007
d	High risk	R1/R2/Rx	52	1049	6.435 (3.427‐12.084)	<.001	5.454 (2.853‐10.425)	<.001
		Tumor multifocal^b^						
I	Low risk	Single	97	NA	1		1	
II	Low risk	Multiple	23	2034	2.233 (1.130‐4.414)	.021	1.705 (0.834‐3.484)	.143
III	High risk	Single	91	1466	2.454 (1.468‐4.100)	.001	2.353 (1.404‐3.944)	.001
IV	High risk	Multiple	26	1366	3.446 (1.734‐6.848)	<.001	3.289 (1.644‐6.579)	.001

Abbreviations: CI, confidence interval; HR, hazard rate; MST, median survival time; NA, not available.

^a^ Information of residual tumor was unavailable in one patients.

^b^ Information of tumor multifocal was unavailable in 20 patients.

^c^ Adjusted for age, residual tumor, and tumor multifocal.

### 3.2 Function exploration of the four prognostic snoRNAs in sarcomas

**Figure 6:**
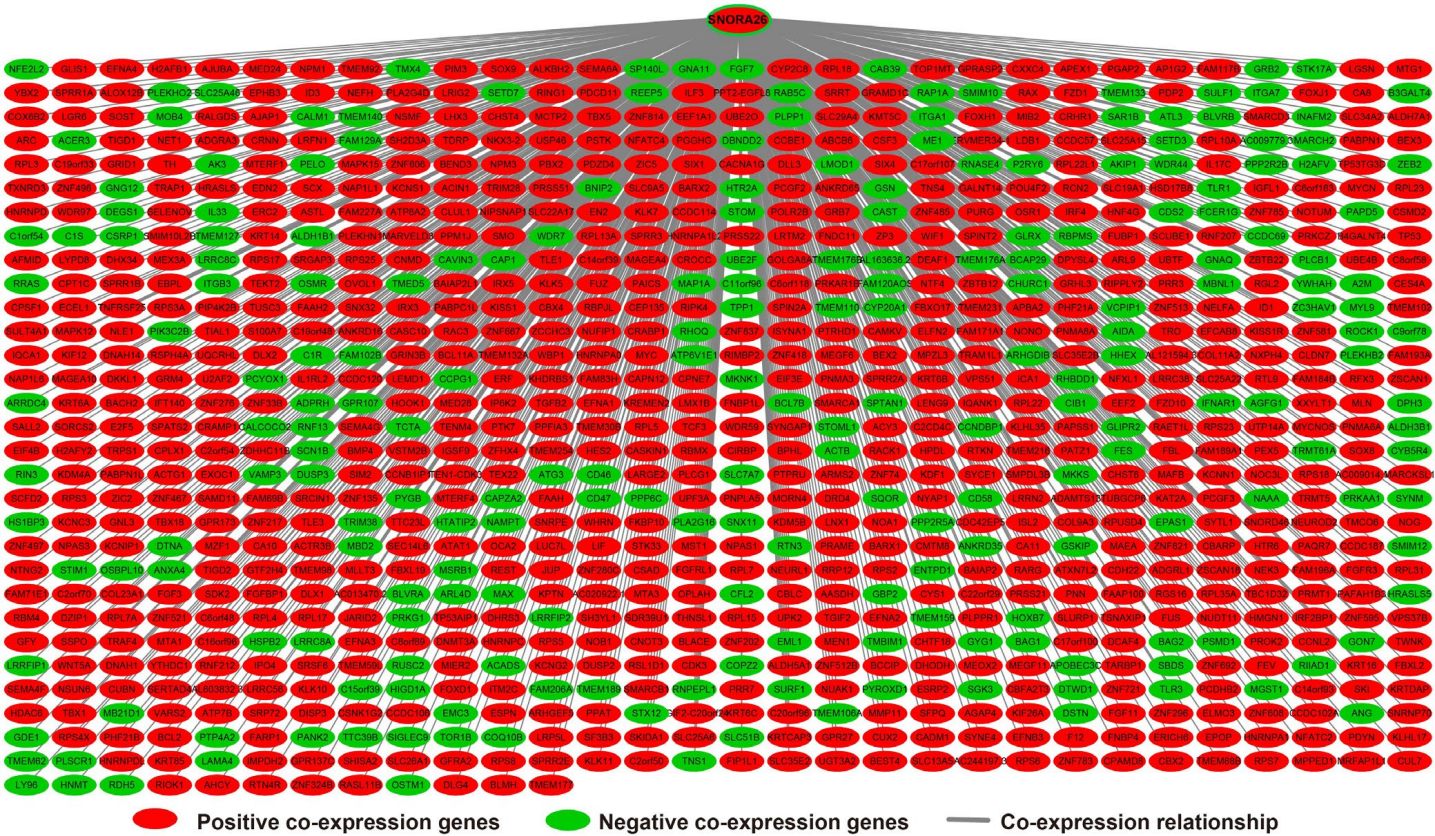
SnoRNA‐genes co‐expression interaction networks of SNORA26

**Figure 7:**
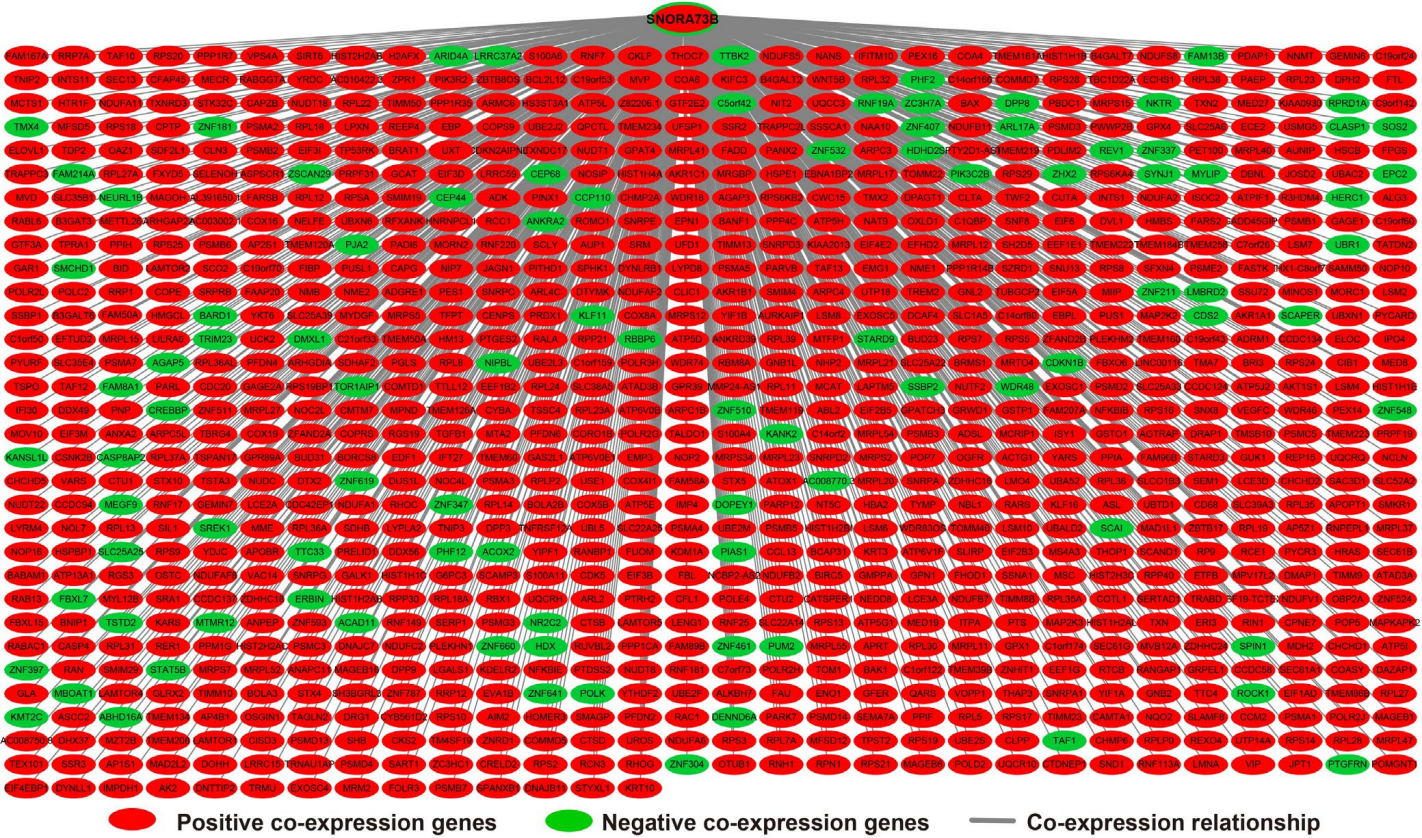
SnoRNA‐genes co‐expression interaction networks of SNORA73B

**Figure 8:**
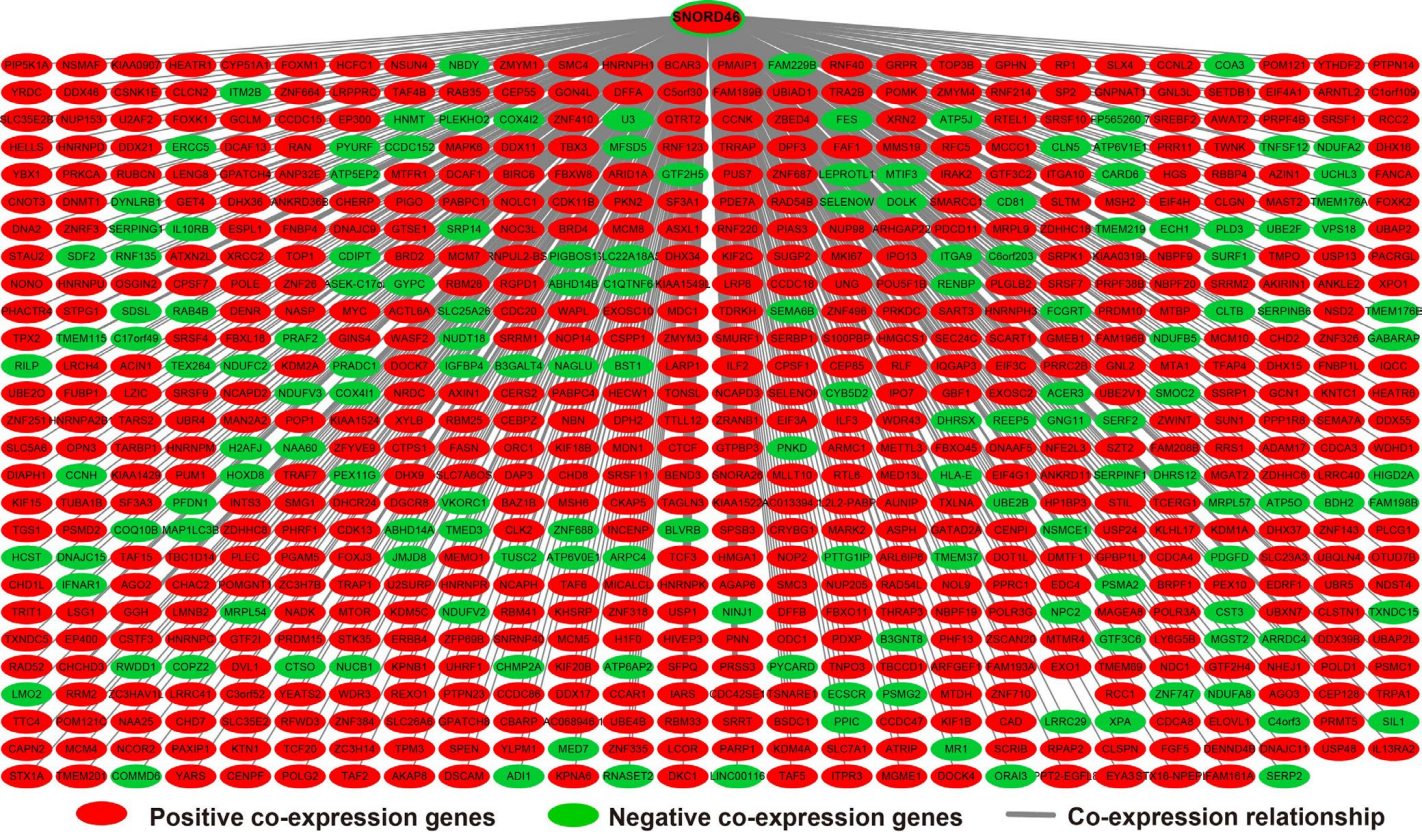
SnoRNA‐genes co‐expression interaction networks of SNORD46

**Figure 9:**
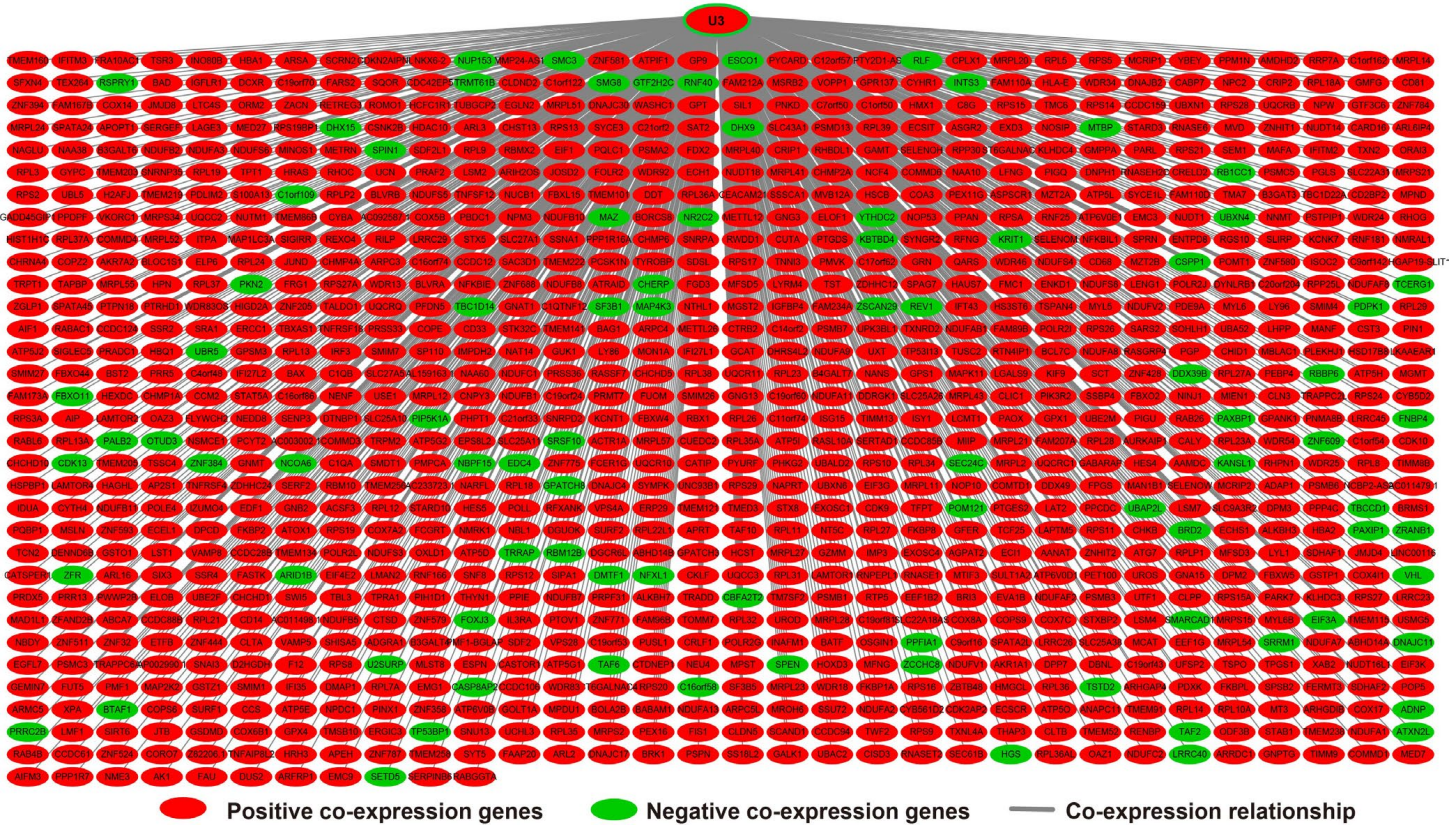
SnoRNA‐genes co‐expression interaction networks of U3

**Figure 10:**
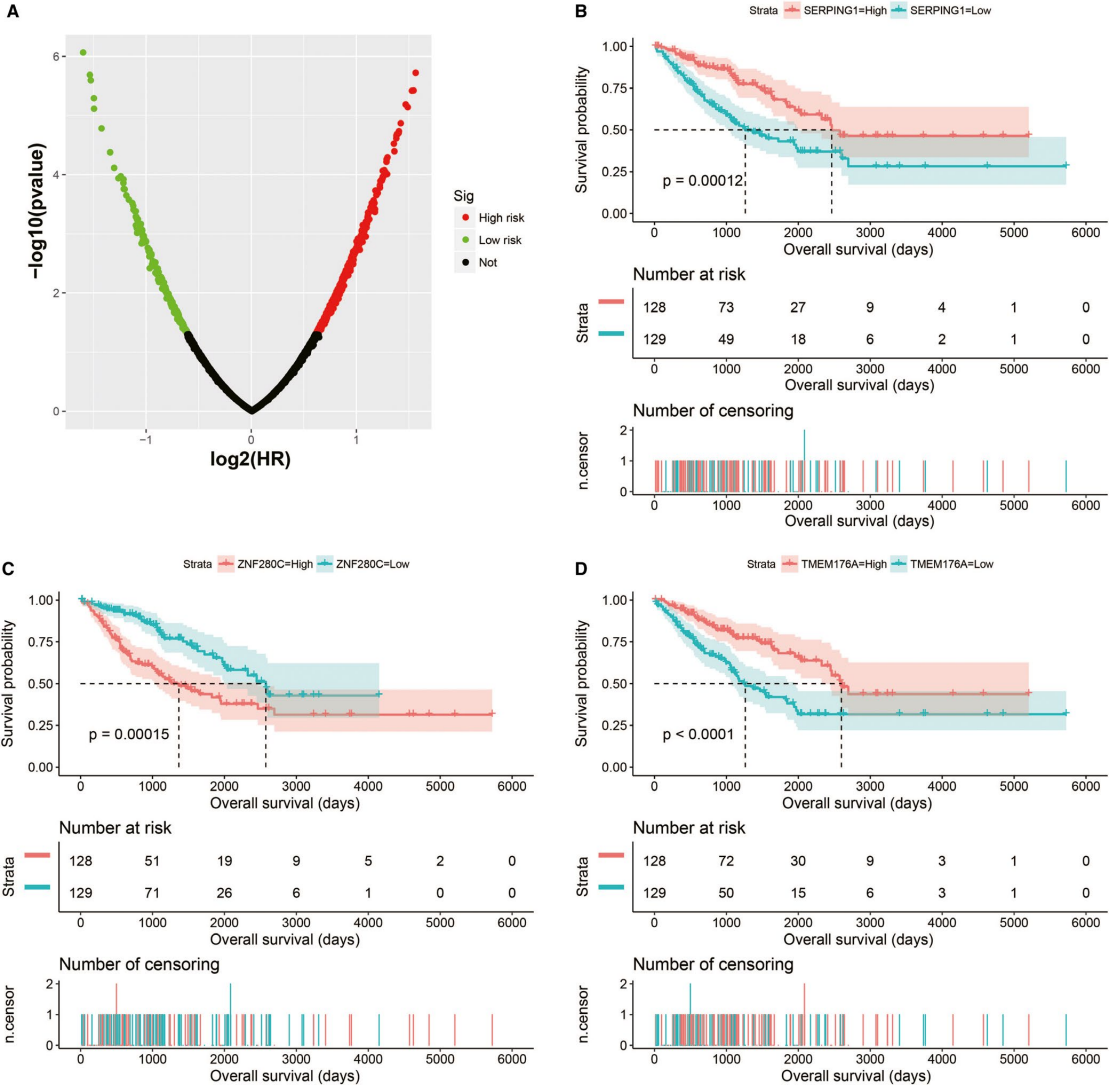
Survival analysis results of the four snoRNAs co‐expression genes in sarcoma. A, Multivariate survival analysis results of SnoRNA co‐expressed genes; B, Kaplan‐Meier curves of SERPING1; C, Kaplan‐Meier curves of ZNF280C; D, Kaplan‐Meier curves of TMEM176A

In order to further understand the potential biological functions of this risk score model, we screened the co‐expressed genes of the four snoRNAs that make up the risk score model, and then functional annotation. A total of 912 genes were markedly co‐expressed with SNORA26 (Figure 6; Table S3), while 944 genes were markedly co‐expressed with SNORA73B (Figure 7; Table S4). There were 699 genes that were markedly co‐expressed with SNORD46 (Figure 8; Table S5), and 1036 genes were markedly co‐expressed with U3 (Figure 9; Table S6). Functional enrichment analysis by DAVID v6.8 using Kyoto Encyclopedia of Genes and Genomes (KEGG) suggests that SNORA26 co‐expression genes notably enriched in pathways in cancer, Rap1, Hippo, mitogen‐activated protein kinase (MAPK), sphingolipid, transforming growth factor beta (TGF‐β), and Wnt signaling pathway (Table S7). Gene Ontology (GO) analysis suggest that SNORA26 co‐expression genes notably enriched in positive regulation of cell proliferation, negative regulation of Wnt signaling pathway, negative regulation of apoptotic process, fibroblast growth factor receptor signaling pathway, cell‐cell signaling, canonical Wnt signaling pathway, cellular response to drug, and Wnt signaling pathway (Table S7). BiNGO analysis also suggest that SNORA26 co‐expression genes notably enriched in cell differentiation, regulation of cell development, regulation of transcription, cell‐cell signaling, and activation of protein kinase B activity (Figure S1). Functional enrichment analysis by DAVID v6.8 suggests that SNORA73B co‐expression genes were markedly enriched in oxidative phosphorylation, metabolic pathways, nuclear factor (NF)‐κB‐inducing kinase (NIK)/NF‐kappaB signaling, stimulatory C‐type lectin receptor signaling pathway, positive regulation of canonical Wnt signaling pathway, T cell receptor signaling pathway, Fc‐epsilon receptor signaling pathway, cell‐cell adhesion, cell‐cell adherens junction, MAPK cascade, cell growth, cell division, positive regulation of target of rapamycin (TOR) signaling, and positive regulation of mitotic cell cycle (Table S8). BiNGO analysis also partly support these results, which markedly enriched in cell cycle, regulation of protein ubiquitination, and RNA metabolic process, (Figure S2). Functional enrichment analysis suggests that SNORD46 co‐expression genes were markedly enriched in mRNA surveillance pathway, DNA replication, mismatch repair, oxidative phosphorylation, cell cycle, DNA repair, cell proliferation, cell division, regulation of cell motility, G1/S transition of mitotic cell cycle, NF‐kappaB binding, cell‐cell adhesion, cell‐cell adherens junction, apoptotic DNA fragmentation, B cell differentiation, negative regulation of autophagy (Table S9). BiNGO analysis also partly support these results, which markedly enriched in regulation of gene expression, DNA replication, DNA repair, cell division, cell cycle, cell proliferation, oxidative phosphorylation, and response to drug (Figure S3). Functional enrichment analysis suggests that U3 co‐expression genes were remarkably enriched in oxidative phosphorylation, metabolic pathways, focal adhesion, NIK/NF‐kappaB signaling, autophagy, cell separation after cytokinesis, apoptotic signaling pathway, MyD88‐independent toll‐like receptor signaling pathway, mitotic metaphase plate congression, positive regulation of TOR signaling, and tumor necrosis factor‐mediated signaling pathway (Table S10). BiNGO analysis also partly support these results, which remarkably enriched in oxidative phosphorylation, regulation of protein ubiquitination, cellular metabolic process, and RNA metabolic process (Figure S4). Subsequently, we combined the co‐expressed genes of four snoRNAs to calculate the prognostic value of these genes in sarcomas. We obtained a total of 2967 co‐expressed genes. Multivariate Cox proportional risk regression model survival analysis suggested that a total of 697 genes were significantly associated with sarcoma prognosis (Table S11). The volcano plot of the prognostic analysis results is summarized in Figure 10A. The top three most significant genes are SERPING1 (log‐rank *P* = .00012, adjusted *P* < .0001, HR = 0.329, 95% CI = 0.211‐0.512, Figure 10B), ZNF280C (log‐rank *P* = .00015, adjusted *P* < .0001, HR = 2.943, 95% CI = 1.887‐4.590, Figure 10C), and TMEM176A (log‐rank *P* < .0001, adjusted *P* < .0001, HR = 0.343, 95% CI = 0.221‐0.534, Figure 10D).

### 3.3 Functional annotation of the risk score

**Figure 11:**
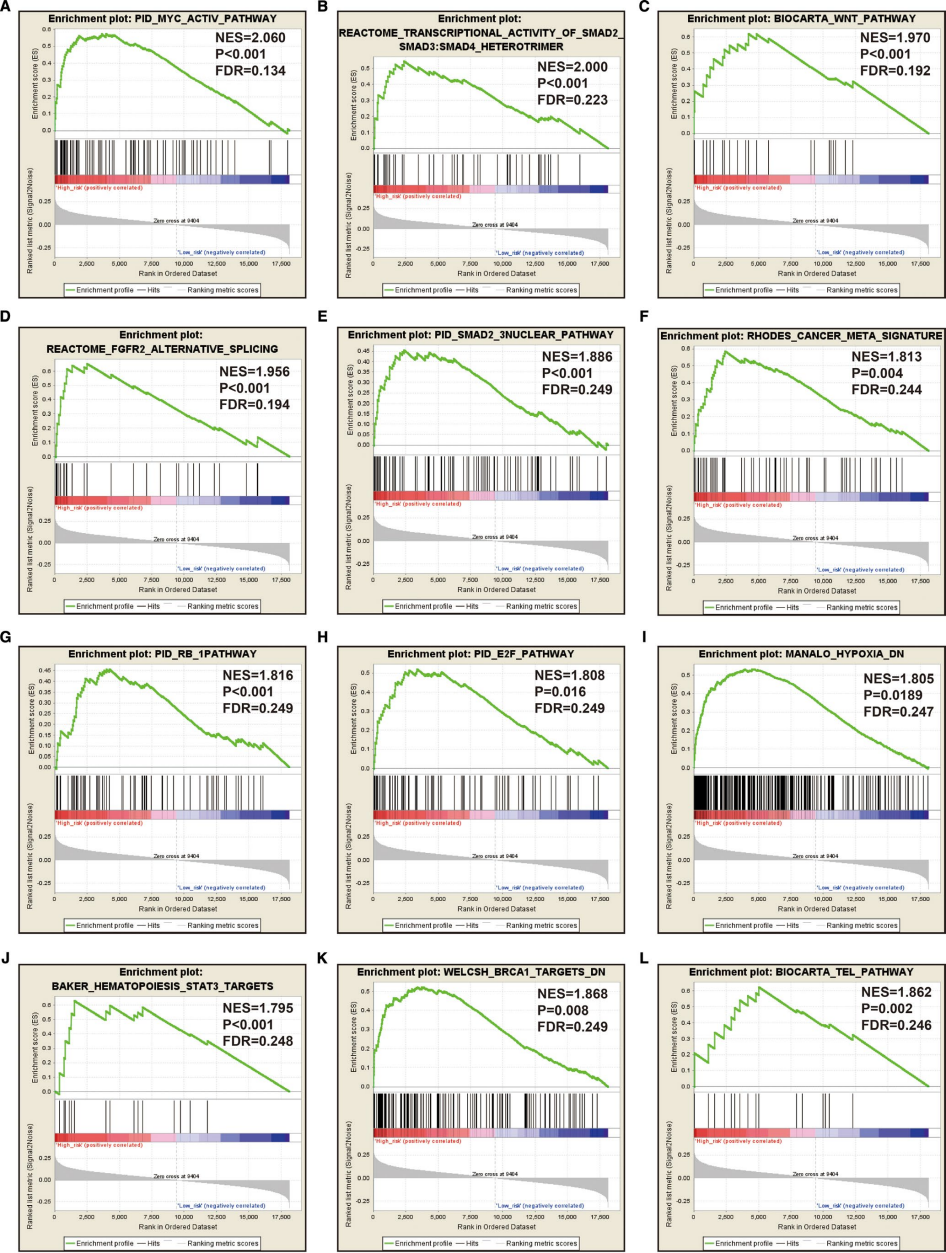
GSEA analysis between low‐ and high‐risk score groups using the c2 reference gene set (c2.all.v7.0.symbols.gmt, A‐L)

**Figure 12:**
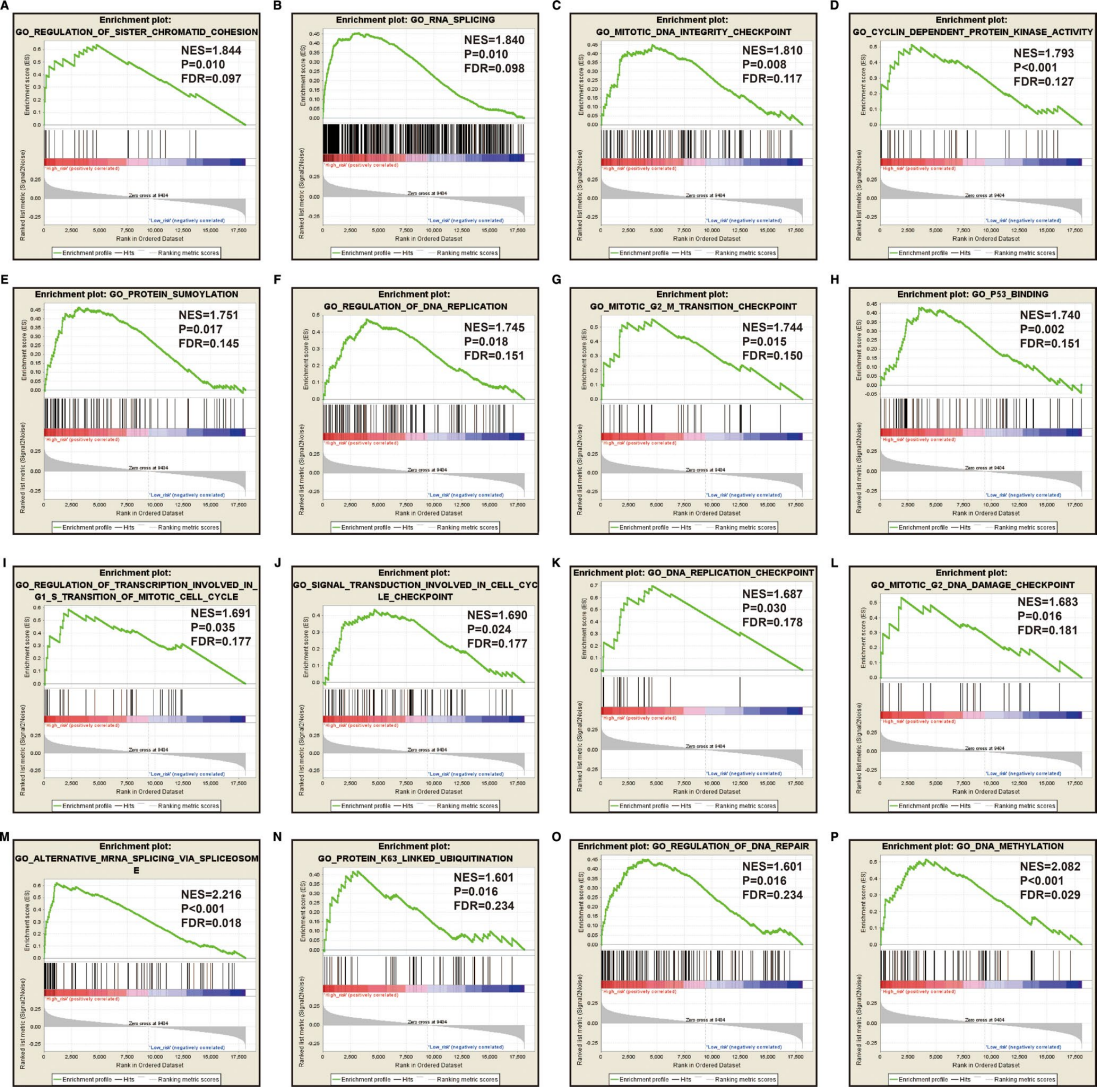
GSEA analysis between low‐ and high‐risk score groups using the c5 reference gene set (c5.all.v7.0.symbols.gmt, A‐P)

**Figure 13:**
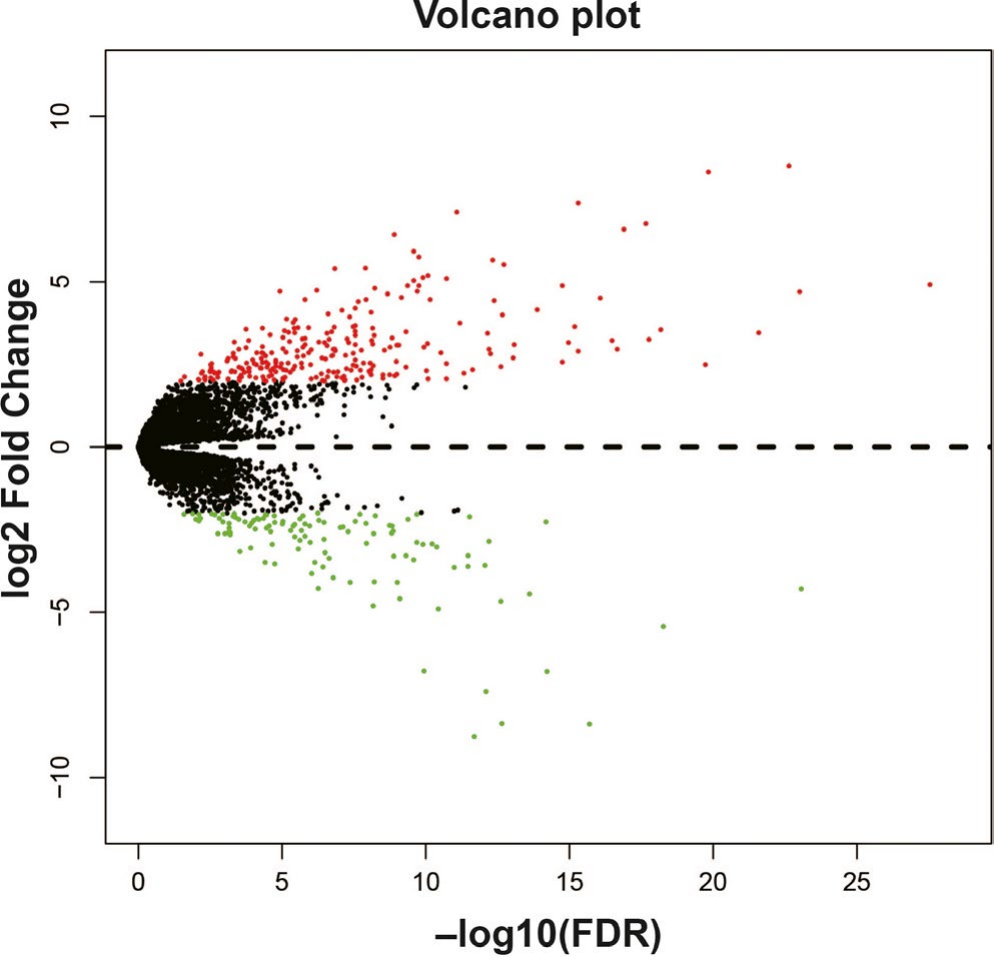
Volcano plot of differentially expressed genes (DEGs) between low‐ and high‐risk score groups. *Notes*: Green nodes represent downregulated DEGs and red nodes represent upregulated DEGs, and the black nodes represent non‐DEGs

**Figure 14:**
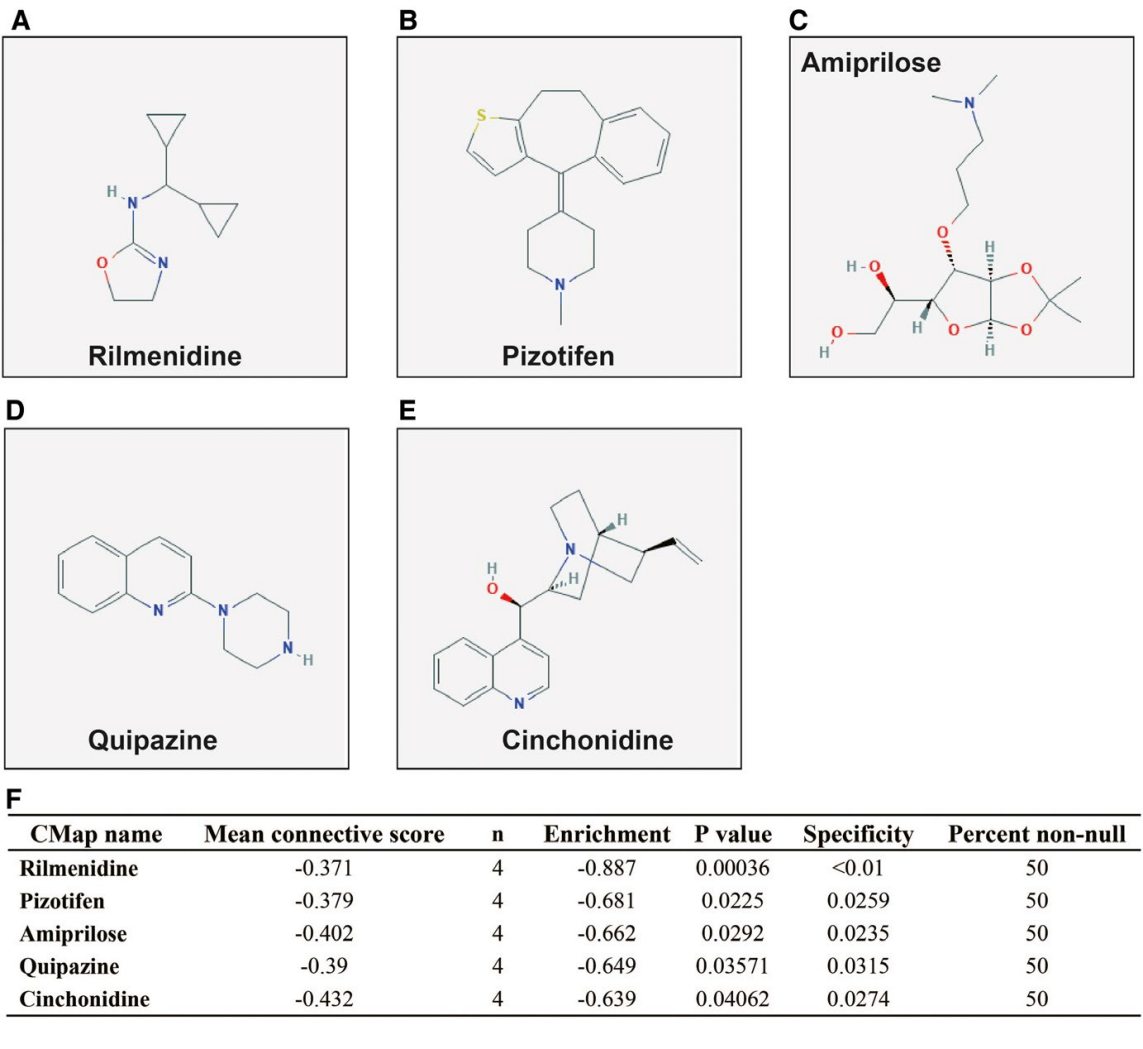
CMap analysis results for low‐ and high‐risk score groups. A, Chemical structure of rilmenidine; B, Chemical structure of pizotifen; C, Chemical structure of amiprilose; D, Chemical structure of quipazine; E, Chemical structure of cinchonidine; F, CMap analysis results

**Figure 15:**
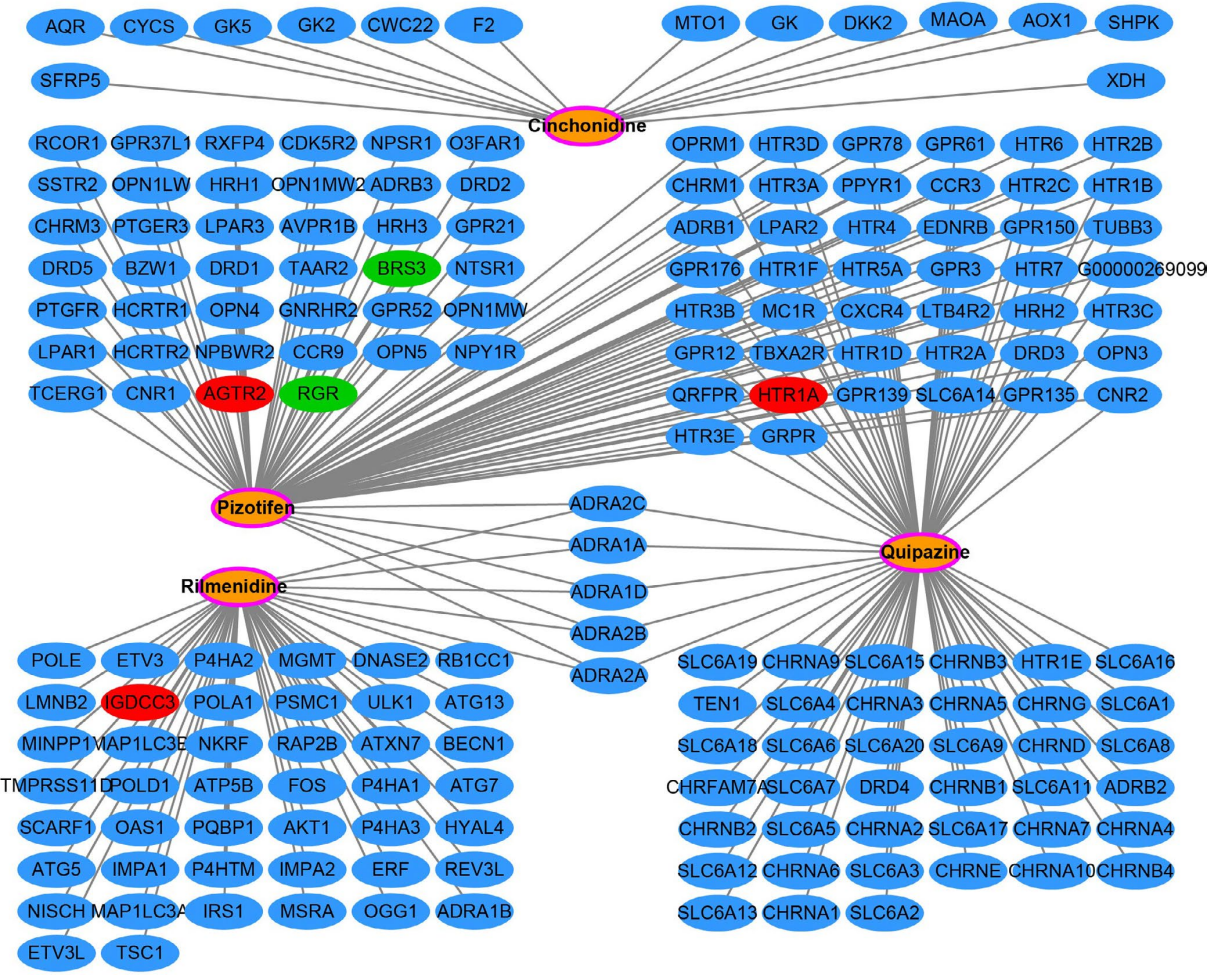
Drug‐gene interaction networks generated from STITCH *Notes*: Green nodes represent downregulated differentially expressed genes (DEGs); red nodes represent upregulated DEGs; the orange nodes with the pink ring represent the drugs; the blue nodes represent the drug interaction genes

Through the above functional enrichment analysis of the snoRNA co‐expressed genes, we have learned about the snoRNAs that constitute the risk score model. In this section, we continue to explore the potential biological function mechanisms of the risk score model. Using the c2 reference gene set as a control gene set for GSEA analysis, we found that high risk score phenotype can be enriched in Myc, Wnt, SMAD2, SMAD3 nuclear, RB1, E2F, and TEL pathways, as well as transcriptional activity of SMAD2, SMAD3, SMAD4 heterotrimer, fibroblast growth factor receptor 2 (FGFR2) alternative splicing, cancer meta signature, hypoxia, BRCA1 targets, and hematopoiesis signal transducer and activator of transcription 3 (STAT3) targets (Figure 11A‐L; Table S12). GSEA analysis using c5 reference gene set suggests that high risk score phenotype can be enriched in cell cycle‐related biological processes, RNA splicing, protein SUMOylation, DNA replication, p53 binding, alternative mRNA splicing via spliceosome, protein k63 linked ubiquitination, regulation of DNA repair, and DNA methylation (Figure 12A‐P; Table S13). In addition, we used genome‐wide RNA‐sequencing (RNA‐seq) dataset to screen for DEGs between patients in the high‐ and low‐risk score groups. We received a total of 371 DEGs between high‐ and low‐risk score group, of them, 113 DEGs were downregulated and 258 DEGs were upregulated (Figure 13; Table S14). The heat map of these DEGs is shown in Figure S5. Functional enrichment analysis of DEGs suggests that these genes were significantly enriched in G protein‐coupled receptor signaling pathway, leukocyte transendothelial migration, renin‐angiotensin system, cellular protein metabolic process, G protein‐coupled receptor binding, calcium‐independent cell‐cell adhesion via plasma membrane cell adhesion molecules, and transmembrane signaling receptor activity (Table S15). The BiNGO analysis result is shown in Figure S6, and indicates that these DEGs were markedly enriched in multicellular organismal development, G protein‐coupled receptor protein signaling pathway, killing of cells of another organism, cell differentiation, muscle cell development, muscle cell differentiation, and tissue development. Then we used these DEGs for CMap analysis, and we obtained a total of five targeted drugs (rilmenidine, pizotifen, amiprilose, quipazine, and cinchonidine) for this risk score in sarcoma. The list of CMap analysis results and the chemical structure of the five drugs are shown in Figure 14A‐F. Subsequently, we used STICH to construct the drug‐gene interaction networks, and we obtained a total of 237 drug‐gene interaction pairs (Figure 15). By analyzing the results of the drug‐gene interaction network and DEG analysis results, we found that rilmenidine can exert a drug effect in sarcomas by interacting with the immunoglobulin superfamily DCC subclass member 3 (IGDCC3) gene. While the pizotifen functions through 5‐hydroxytryptamine receptor 1A (HTR1A), angiotensin II receptor type 2 (AGTR2), retinal G protein‐coupled receptor (RGR), and bombesin receptor subtype 3 (BRS3) genes, as well as quipazine functions through HTR1A gene (Figure 15).

## 4 DISCUSSION

Recently, more and more evidences show that snoRNA plays an important role in cancers and can be used as tumor biomarker. A study by Zhao and his co‐workers have reported that they screened prognostic snoRNAs using clear cell renal cell carcinoma (ccRCC) patients in the TCGA cohort, and also established a six‐snoRNA prognostic signature, which can divide patients into high‐risk and low‐risk groups, and through prognostic analysis and independent cohort verification, it was found that high‐risk patients had a poor prognosis. After evaluating, the prediction accuracy of this prognostic signature is higher than traditional clinical parameters. At the same time, they also determined that the prognostic signature is also highly accurate and specific in ccRCC diagnosis.^28^ Xing et al screened out 113 prognostic snoRNAs by performing univariate survival analysis in TCGA head and neck squamous cell carcinoma (HNSCC) cohort. They then constructed a five‐snoRNA signature using the least absolute shrinkage and selection operator (LASSO) method to divide HNSCC into different subgroups and prognostic risk patients.^29^ In the present study, we also identified 15 prognostic snoRNAs, and identified a four‐snoRNA signature for sarcoma overall survival. Through this study, we also screened snoRNA for prognosis in sarcoma patients for the first time, confirming that snoRNA expression level can be used to predict the prognosis of sarcoma. This is consistent with previous research results. Meanwhile, these four snoRNAs have never been reported to be significantly correlated with the prognosis of sarcomas in previous studies. This study is the first to report these four prognostic snoRNAs of sarcomas.

For the functional mechanism of these four snoRNAs, we use protein‐coding genes that are significantly co‐expressed with snoRNA for functional annotation, which were the same as the previous study.^29^ We have noticed that the co‐expressed genes of these four prognostic snoRNAs are significantly enriched in some classical biological functions and pathways that are significantly related to the basic functional state of the cell and cancers. Functional enrichment suggest that the co‐expressed genes of SNORA26 can be significantly enriched into Rap1 signaling pathway, pathways in cancer, Hippo signaling pathway, MAPK signaling pathway, sphingolipid signaling pathway, TGF‐β signaling pathway and Wnt signaling pathway. The above enrichment results have been reported to be closely related to sarcomas in previous studies. Liu et al found that in osteosarcoma, circular RNA can participate in the occurrence and development of osteosarcoma by participating in regulating the Rap1 signaling pathway.^30^ Rap1 signaling pathway was also found to be significantly associated with lung metastasis of osteosarcoma.^31^ Our previous study also reported that long noncoding RNA plasmacytoma variant translocation 1 can function in sarcoma through Wnt signaling pathway.^32^ Drugs or specific genes can affect the malignant phenotype of osteosarcoma by regulating the MAPK signaling pathway, indicating that MAPK can be used as a target for targeted therapy in osteosarcoma.^33^, ^34^, ^35^ Bone morphogenic protein and TGFB signaling pathways play an important role in the development of central chondrosarcoma and osteosarcoma, suggesting that TGFB inhibitors can be used in targeted therapy of sarcoma.^36^, ^37^ Functional enrichment analysis suggests that SNORA73B was significantly enriched in NF‐κB, TOR, Wnt signaling pathway, MAPK cascade, cell adhesion, and cell cycle. Cell cycle and cell adhesion are the basic state of tumor cells, and are closely related to tumor proliferation and metastasis, respectively. High expression of TOR signaling pathway suggested that the sarcoma was highly invasive and significantly correlated with poor prognosis of the sarcoma, which all suggested that it could be used as a targeted therapeutic target for the sarcoma.^38^ Targeted regulation of the TOR signaling pathway by drugs can affect the malignant phenotype of sarcomas.^39^, ^40^ NF‐κB has been reported as a biomarker for sarcoma treatment efficacy and differentiation.^41^ Functional enrichment analysis suggests that SNORD46 and U3 were significantly enriched in NF‐κB signaling pathway, cell adhesion, and cell cycle, which function was similar to the above two snoRNAs.

Among snoRNA co‐expressed genes, we found that SERPING1 is the core gene in the tumorigenesis of osteosarcoma,^42^ while it was significantly correlated with the sarcoma overall survival in the present study. For the GSEA analysis of the risk score, we found that the high risk phenotype was correlated with Myc, Wnt, RB1, E2F, and TEL signaling pathways, STAT3 and BRCA1 target, cell cycle‐related biological processes, RNA splicing, protein SUMOylation, DNA replication, p53 binding, DNA repair, and DNA methylation. Schwentner et al found that E2F factors are inhibited in Ewing sarcoma, which can activate the abnormal cell cycle of cancer.^43^ Cote et al through next‐generation sequencing of sarcoma found that most common mutations of sarcomas were in the cell cycle, including RB1 mutations.^44^ Liu et al study proved that Rb1 family mutation is sufficient for sarcoma initiation.^45^ Cheng et al reported that copy number variation signature of Myc can be used as chemotherapy‐response biomarkers in pediatric sarcoma.^46^ Liu et al found that apatinib can promote osteosarcoma autophagy and apoptosis through STAT3, which suggests that STAT3 may be the target of osteosarcoma therapy.^47^ At the same time, the phosphorylation of STAT3 is significantly associated with the prognosis of undifferentiated pleomorphic sarcoma.^48^ BRCA1 and p53 have also been reported to be associated with sarcomas in previous studies.^49^, ^50^, ^51^, ^52^

For CMap analysis, we identified five drugs for the risk score model in sarcoma. In previous studies, pizotifen has been reported to be closely associated with cancers, and can be used as an anticancer drug. Pizotifen can significantly inhibit colon and gastric cancer cell malignant phenotype via suppressing Wnt signaling pathway.^53^, ^54^ Vucicevic et al found that rilmenidine‐derived compounds can synergize the antitumor effects of doxorubicin.^55^ Srdic‐Rajic and his co‐workers found that rilmenidine can inhibit leukemia cell proliferation and promote apoptosis through the mitochondrial pathway.^56^ By reviewing the literature, we did not find that quipazine, amiprilose, and cinchonidine drugs were reported to be significantly correlated with cancers in previous studies.

There are still some limitations to be noted in this study. Firstly, this study is a single‐center study based on the TCGA sarcoma cohort. In the future, we still need multicenter large cohort data to verify our findings. Secondly, the data of this study were obtained from the TCGA website. The obtained clinical parameter information is incomplete. There may be some clinical parameters that affect the prognosis of sarcoma that are not included in the prognostic analysis model of this study, which leads to deviations in our results. Thirdly, because the results of our study were derived from RNA‐seq dataset and bioinformatics analysis methods, the drugs and functional mechanisms we screened were not verified by in vivo or in vitro experiments, and our results still need further experimental verification in future studies. Despite the limitations mentioned above in this study, our study is the first to report the screening of snoRNA prognostic markers for sarcoma from RNA‐seq datasets, which provide theoretical basis for further understanding the clinical application value of snoRNA in sarcoma. At the same time, the snoRNA prognostic markers screened in this study and the constructed risk score model are also expected to be applied in clinical practice in the future.

## 5 CONCLUSIONS

In this study, we identified 15 snoRNAs that were markedly related to the prognosis of sarcoma using the RNA‐seq dataset of the TCGA sarcoma cohort, and constructed a prognostic signature based on four prognostic snoRNA expression values (U3, SNORA73B, SNORD46, and SNORA26). We also annotated the functions of the four snoRNAs by their co‐expression genes, and found that some of them were closely related to cell cycle‐related biological processes and tumor‐related signaling pathways, such as Wnt, MAPK, TOR, and NF‐kappa B signaling pathway. GSEA of the risk score suggests that high risk score phenotype was markedly enriched in cell cycle‐related biological processes, protein SUMOylation, DNA replication, p53 binding, regulation of DNA repair, and DNA methylation, as well as Myc, Wnt, RB1, E2F, and TEL pathways. Then, we also used the CMap online tool to screen the five targeted drugs (rilmenidine, pizotifen, amiprilose, quipazine, and cinchonidine) for this risk score model in sarcoma. Our current study is the first comprehensive investigation of the clinical application value and potential functional mechanism of snoRNAs in sarcoma through whole‐genome RNA‐seq dataset. Since our research lacks validation in vivo and in vitro experiments, our findings still need to be further validated in future studies.

## CONFLICT OF INTEREST

The authors have declared that no competing interest exists.

## AUTHOR CONTRIBUTIONS

Jianwei Liu and Rong Li designed the study; Jianwei Liu, Xiwen Liao, Xianze Zhu, and Peizhen Lv are responsible for the collection, statistics, and interpretation of data; Rong Li is responsible for critical review; Jianwei Liu drafted the manuscript; and Rong Li revised the manuscript. All authors approved the final manuscript.

## Supporting information

Fig S1

Fig S2

Fig S3

Fig S4

Fig S5

Fig S6

Table S1‐S15

## Data Availability

The data that support the findings of this study are openly available in TCGA (https://cancergenome.nih.gov/).

## References

[1] Ceyssens S , Stroobants S . Sarcoma. Methods Mol Biol. 2011;727:191‐203. 21331935 10.1007/978-1-61779-062-1_11

[2] Ferrari A , Dirksen U , Bielack S . Sarcomas of soft tissue and bone. Prog Tumor Res. 2016;43:128‐141. 27595362 10.1159/000447083

[3] Skubitz KM , D'Adamo DR . Sarcoma. Mayo Clin Proc. 2007;82:1409‐1432. 17976362 10.4065/82.11.1409

[4] Gilbert NF , Cannon CP , Lin PP , Lewis VO . Soft‐tissue sarcoma. J Am Acad Orthop Surg. 2009;17:40‐47. 19136426 10.5435/00124635-200901000-00006

[5] Xing YH , Chen LL . Processing and roles of snoRNA‐ended long noncoding RNAs. Crit Rev Biochem Mol Biol. 2018;53:596‐606. 30252509 10.1080/10409238.2018.1508411

[6] Liang J , Wen J , Huang Z , Chen XP , Zhang BX , Chu L . Small nucleolar RNAs: insight into their function in cancer. Front Oncol. 2019;9:587. 31338327 10.3389/fonc.2019.00587 PMC6629867

[7] Williams GT , Farzaneh F . Are snoRNAs and snoRNA host genes new players in cancer? Nat Rev Cancer. 2012;12:84‐88. 22257949 10.1038/nrc3195

[8] Gong J , Li Y , Liu C‐J , et al. A pan‐cancer analysis of the expression and clinical relevance of small nucleolar RNAs in Human Cancer. Cell Rep. 2017;21:1968‐1981. 29141226 10.1016/j.celrep.2017.10.070

[9] Romano G , Veneziano D , Acunzo M , Croce CM . Small non‐coding RNA and cancer. Carcinogenesis. 2017;38:485‐491. 28449079 10.1093/carcin/bgx026 PMC6248440

[10] Swiatowy W , Jagodzińśki PP . Molecules derived from tRNA and snoRNA: Entering the degradome pool. Biomed Pharmacother. 2018;108:36‐42. 30216797 10.1016/j.biopha.2018.09.017

[11] Cancer Genome Atlas Research Network , Weinstein JN , Collisson EA , et al. 2013. The Cancer Genome Atlas Pan‐Cancer analysis project. Nat Genet. 45:1113‐1120. 24071849 10.1038/ng.2764 PMC3919969

[12] Cancer Genome Atlas Research Network . Comprehensive and integrated genomic characterization of adult soft tissue sarcomas. Cell. 2017;171(950–965):e928. 10.1016/j.cell.2017.10.014 PMC5693358 29100075

[13] Robinson MD , McCarthy DJ , Smyth GK . edgeR: a Bioconductor package for differential expression analysis of digital gene expression data. Bioinformatics. 2010;26:139‐140. 19910308 10.1093/bioinformatics/btp616 PMC2796818

[14] Liao X , Wang X , Huang K , et al. Integrated analysis of competing endogenous RNA network revealing potential prognostic biomarkers of hepatocellular carcinoma. J Cancer. 2019;10:3267‐3283. 31289599 10.7150/jca.29986 PMC6603367

[15] Liao X , Zhu G , Huang R , et al. Identification of potential prognostic microRNA biomarkers for predicting survival in patients with hepatocellular carcinoma. Cancer Manag Res. 2018;10:787‐803. 29713196 10.2147/CMAR.S161334 PMC5912208

[16] Liao X , Liu X , Yang C , et al. Distinct diagnostic and prognostic values of minichromosome maintenance gene expression in patients with hepatocellular carcinoma. J Cancer. 2018;9:2357‐2373. 30026832 10.7150/jca.25221 PMC6036720

[17] Luo SS , Liao XW , Zhu XD . Genome‐wide analysis to identify a novel microRNA signature that predicts survival in patients with stomach adenocarcinoma. J Cancer. 2019;10:6298‐6313. 31772663 10.7150/jca.33250 PMC6856753

[18] da Huang W , Sherman BT , Lempicki RA . Systematic and integrative analysis of large gene lists using DAVID bioinformatics resources. Nat Protoc. 2009;4:44‐57. 19131956 10.1038/nprot.2008.211

[19] Maere S , Heymans K , Kuiper M . BiNGO: a Cytoscape plugin to assess overrepresentation of gene ontology categories in biological networks. Bioinformatics. 2005;21:3448‐3449. 15972284 10.1093/bioinformatics/bti551

[20] Subramanian A , Tamayo P , Mootha VK , et al. Gene set enrichment analysis: a knowledge‐based approach for interpreting genome‐wide expression profiles. Proc Natl Acad Sci USA. 2005;102:15545‐15550. 16199517 10.1073/pnas.0506580102 PMC1239896

[21] Lamb J , Crawford ED , Peck D , et al. The Connectivity Map: using gene‐expression signatures to connect small molecules, genes, and disease. Science. 2006;313:1929‐1935. 17008526 10.1126/science.1132939

[22] Lamb J . The Connectivity Map: a new tool for biomedical research. Nat Rev Cancer. 2007;7:54‐60. 17186018 10.1038/nrc2044

[23] Kim S , Thiessen PA , Cheng T , et al. Literature information in PubChem: associations between PubChem records and scientific articles. J Cheminform. 2016;8:32. 27293485 10.1186/s13321-016-0142-6 PMC4901473

[24] Kuhn M , Szklarczyk D , Pletscher‐Frankild S , et al. STITCH 4: integration of protein‐chemical interactions with user data. Nucleic Acids Res. 2014;42:D401‐D407. 24293645 10.1093/nar/gkt1207 PMC3964996

[25] Kuhn M , Szklarczyk D , Franceschini A , von Mering C , Jensen LJ , Bork P . STITCH 3: zooming in on protein‐chemical interactions. Nucleic Acids Res. 2012;40:D876‐D880. 22075997 10.1093/nar/gkr1011 PMC3245073

[26] Szklarczyk D , Santos A , von Mering C , Jensen LJ , Bork P , Kuhn M . STITCH 5: augmenting protein‐chemical interaction networks with tissue and affinity data. Nucleic Acids Res. 2016;44:D380‐D384. 26590256 10.1093/nar/gkv1277 PMC4702904

[27] Reiner A , Yekutieli D , Benjamini Y . Identifying differentially expressed genes using false discovery rate controlling procedures. Bioinformatics. 2003;19:368‐375. 12584122 10.1093/bioinformatics/btf877

[28] Zhao Y , Yan Y , Ma R , et al. Expression signature of six‐snoRNA serves as novel non‐invasive biomarker for diagnosis and prognosis prediction of renal clear cell carcinoma. J Cell Mol Med. 2020;24:2215‐2228. 31943775 10.1111/jcmm.14886 PMC7011154

[29] Xing L , Zhang X , Zhang X , Tong D . Expression scoring of a small‐nucleolar‐RNA signature identified by machine learning serves as a prognostic predictor for head and neck cancer [published online ahead of print January14, 2020]. J Cell Physiol. 10.1002/jcp.29462 PMC7540035 31943178

[30] Liu W , Zhang J , Zou C , et al. Microarray expression profile and functional analysis of circular RNAs in osteosarcoma. Cell Physiol Biochem. 2017;43:969‐985. 28957794 10.1159/000481650

[31] Shi Z , Zhou H , Pan B , et al. Exploring the key genes and pathways of osteosarcoma with pulmonary metastasis using a gene expression microarray. Mol Med Rep. 2017;16:7423‐7431. 28944885 10.3892/mmr.2017.7577 PMC5865874

[32] Liu J , Li R , Liao X , Hu B , Yu J . Comprehensive investigation of the clinical significance and molecular mechanisms of plasmacytoma variant translocation 1 in sarcoma using genome‐wide RNA sequencing data. J Cancer. 2019;10:4961‐4977. 31598169 10.7150/jca.31675 PMC6775530

[33] Hsu MJ , Peng SF , Chueh FS , et al. Lupeol suppresses migration and invasion via p38/MAPK and PI3K/Akt signaling pathways in human osteosarcoma U‐2 OS cells. Biosci Biotechnol Biochem. 2019;83:1729‐1739. 31010399 10.1080/09168451.2019.1606693

[34] Liu B , Zhao H , Zhang L , Shi X . Silencing of long‐non‐coding RNA ANCR suppresses the migration and invasion of osteosarcoma cells by activating the p38MAPK signalling pathway. BMC Cancer. 2019;19:1112. 31727012 10.1186/s12885-019-6335-4 PMC6857278

[35] Burotto M , Chiou VL , Lee JM , Kohn EC . The MAPK pathway across different malignancies: a new perspective. Cancer. 2014;120:3446‐3456. 24948110 10.1002/cncr.28864 PMC4221543

[36] Boeuf S , Bovée JVMG , Lehner B , et al. BMP and TGFbeta pathways in human central chondrosarcoma: enhanced endoglin and Smad 1 signaling in high grade tumors. BMC Cancer. 2012;12:488. 23088614 10.1186/1471-2407-12-488 PMC3495847

[37] Yang R , Piperdi S , Zhang Y , et al. Transcriptional profiling identifies the signaling axes of IGF and transforming growth factor‐b as involved in the pathogenesis of osteosarcoma. Clin Orthop Relat Res. 2016;474:178‐189. 26463566 10.1007/s11999-015-4578-1 PMC4686509

[38] Li YX , Ding SS , Wen WJ , Han L , Wang HQ , Shi HY . Impact of the activation status of the Akt/mTOR signalling pathway on the clinical behaviour of synovial sarcoma: retrospective analysis of 174 patients at a single institution. Cancer Manag Res. 2020;12:1759‐1769. 32210617 10.2147/CMAR.S228578 PMC7074818

[39] Martin SB , Reiche WS , Fifelski NA , et al. Leucine and branched‐chain amino acid metabolism contribute to the growth of bone sarcomas by regulating AMPK and mTORC1 signaling. Biochem J. 2020;477:1579‐1599. 32297642 10.1042/BCJ20190754

[40] Zhang H , Jiang H , Zhang H , Liu J , Hu X , Chen L . Anti‐tumor efficacy of phellamurin in osteosarcoma cells: involvement of the PI3K/AKT/mTOR pathway. Eur J Pharmacol. 2019;858:172477. 31228450 10.1016/j.ejphar.2019.172477

[41] Rivera‐Reyes A , Ye S , Marino GE , et al. YAP1 enhances NF‐kappaB‐dependent and independent effects on clock‐mediated unfolded protein responses and autophagy in sarcoma. Cell Death Dis. 2018;9:1108. 30382078 10.1038/s41419-018-1142-4 PMC6208433

[42] Pan Y , Lu L , Chen J , Zhong Y , Dai Z . Identification of potential crucial genes and construction of microRNA‐mRNA negative regulatory networks in osteosarcoma. Hereditas. 2018;155:21. 29760609 10.1186/s41065-018-0061-9 PMC5941338

[43] Schwentner R , Papamarkou T , Kauer MO , et al. EWS‐FLI1 employs an E2F switch to drive target gene expression. Nucleic Acids Res. 2015;43:2780‐2789. 25712098 10.1093/nar/gkv123 PMC4357724

[44] Cote GM , He J , Choy E . Next‐generation sequencing for patients with sarcoma: a single center experience. Oncologist. 2018;23:234‐242. 28860410 10.1634/theoncologist.2017-0290 PMC5813739

[45] Liu Y , Sánchez‐Tilló E , Lu X , et al. Rb1 family mutation is sufficient for sarcoma initiation. Nat Commun. 2013;4:2650. 24150016 10.1038/ncomms3650

[46] Cheng L , Pandya PH , Liu E , et al. Integration of genomic copy number variations and chemotherapy‐response biomarkers in pediatric sarcoma. BMC Med Genomics. 2019;12:23. 30704460 10.1186/s12920-018-0456-5 PMC6357363

[47] Liu K , Ren T , Huang YI , et al. Apatinib promotes autophagy and apoptosis through VEGFR2/STAT3/BCL‐2 signaling in osteosarcoma. Cell Death Dis. 2017;8:e3015. 28837148 10.1038/cddis.2017.422 PMC5596600

[48] Bekki H , Kohashi K , Yamada Y , et al. Phosphorylation of STAT3 in undifferentiated pleomorphic sarcoma is correlated with a favorable prognosis. Pathobiology. 2017;84:161‐169. 27652596 10.1159/000448524

[49] Veeraraghavan J , Natarajan M , Herman TS , Aravindan N . Curcumin‐altered p53‐response genes regulate radiosensitivity in p53‐mutant Ewing's sarcoma cells. Anticancer Res. 2010;30:4007‐4015. 21036715

[50] Kappler M , Taubert H , Bartel F , et al. Radiosensitization, after a combined treatment of survivin siRNA and irradiation, is correlated with the activation of caspases 3 and 7 in a wt‐p53 sarcoma cell line, but not in a mt‐p53 sarcoma cell line. Oncol Rep. 2005;13:167‐172. 15583820

[51] Italiano A , Touati N , Litiere S , Collin F , Pourquier P , Gronchi A . Prospective assessment of the predictive value of the BRCA1 gene status in sarcoma patients treated with trabectedin: an updated analysis of the EORTC 62091 trial. Cancer Med. 2018;7:1575‐1577. 29656586 10.1002/cam4.1403 PMC5943428

[52] Gorthi A , Romero JC , Loranc E , et al. EWS‐FLI1 increases transcription to cause R‐loops and block BRCA1 repair in Ewing sarcoma. Nature. 2018;555:387‐391. 29513652 10.1038/nature25748 PMC6318124

[53] Jiang Y , Wang W , Wu X , Shi J . Pizotifen inhibits the proliferation and invasion of gastric cancer cells. Exp Ther Med. 2020;19:817‐824. 32010241 10.3892/etm.2019.8308 PMC6966152

[54] Piao SS , Shang B . Pizotifen inhibits the proliferation and migration of colon cancer HCT116 Cells by down‐regulating Wnt signaling pathway. Ann Clin Lab Sci. 2019;49:183‐188. 31028062

[55] Vucicevic J , Srdic‐Rajic T , Pieroni M , et al. A combined ligand‐ and structure‐based approach for the identification of rilmenidine‐derived compounds which synergize the antitumor effects of doxorubicin. Bioorg Med Chem. 2016;24:3174‐3183. 27265687 10.1016/j.bmc.2016.05.043

[56] Srdic‐Rajic T , Nikolic K , Cavic M , et al. Rilmenidine suppresses proliferation and promotes apoptosis via the mitochondrial pathway in human leukemic K562 cells. Eur J Pharm Sci. 2016;81:172‐180. 26598394 10.1016/j.ejps.2015.10.017

