# Supplementary figures and images for: Identification of potential prognostic small nucleolar RNA biomarkers for predicting overall survival in patients with sarcoma
Source: Cancer Med. 2020 Aug 11;9(19):7018–33. doi: 10.1002/cam4.3361 (PMC7541128; doi:10.1002/cam4.3361)

**<5.00E-7**

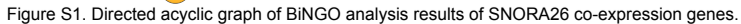

Supplement: Supplementary file 1 — Fig S1 [file CAM4-9-7018-s001.pdf]

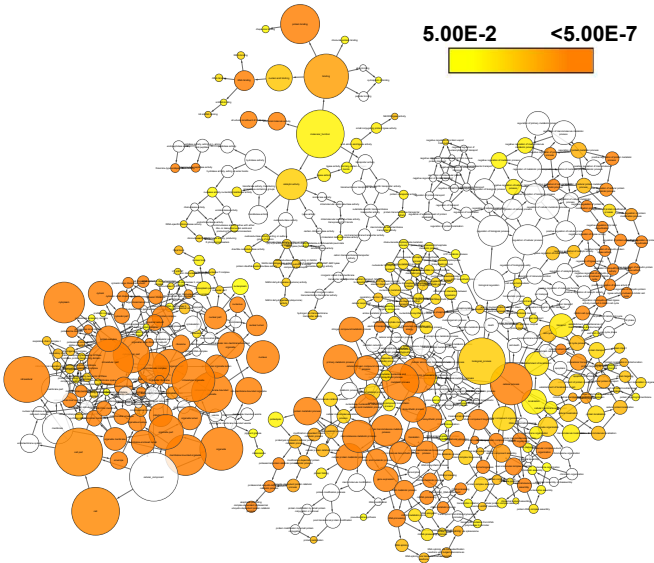

Figure S2. Directed acyclic graph of BiNGO analysis results of SNORA73B co-expression genes.

Supplement: Supplementary file 2 — Fig S2 [file CAM4-9-7018-s002.pdf]

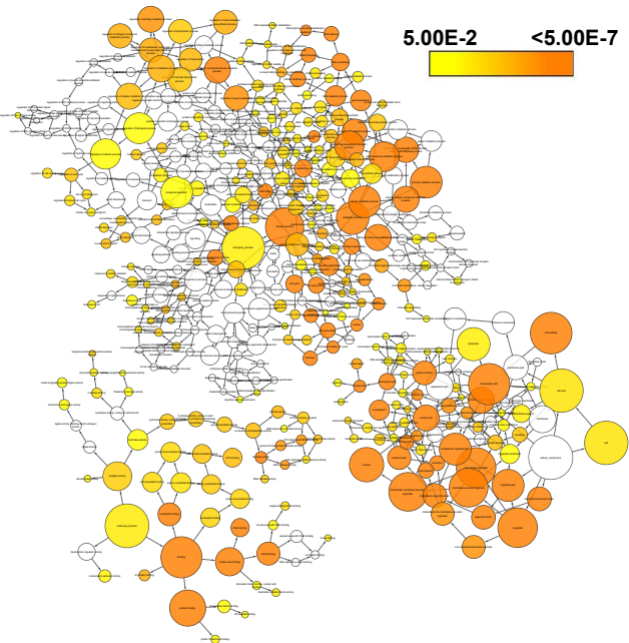

Supplement: Supplementary file 3 — Fig S3 [file CAM4-9-7018-s003.pdf]
