# Supplementary material for: Identification of potential prognostic small nucleolar RNA biomarkers for predicting overall survival in patients with sarcoma
Source: Cancer Med. 2020 Aug 11;9(19):7018–33. doi: 10.1002/cam4.3361 (PMC7541128; doi:10.1002/cam4.3361)
Supplement: Supplementary file 4 — Fig S4 [file CAM4-9-7018-s004.pdf]

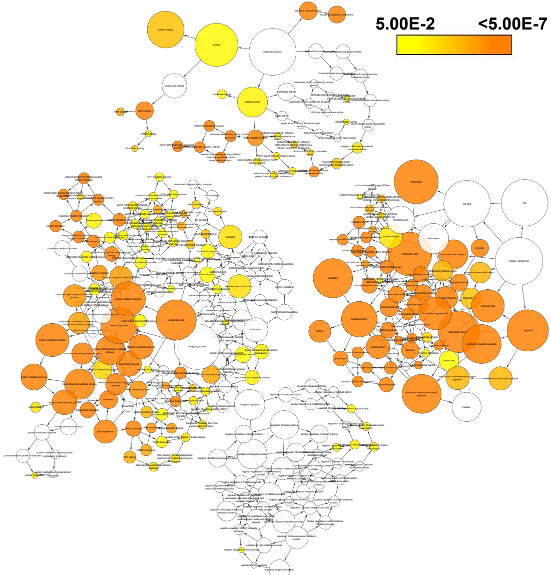

Figure S4. Directed acyclic graph of BiNGO analysis results of U3 co-expression genes.
