# Supplementary material for: Identification of potential prognostic small nucleolar RNA biomarkers for predicting overall survival in patients with sarcoma
Source: Cancer Med. 2020 Aug 11;9(19):7018–33. doi: 10.1002/cam4.3361 (PMC7541128; doi:10.1002/cam4.3361)
Supplement: Supplementary file 6 — Fig S6 [file CAM4-9-7018-s006.pdf]

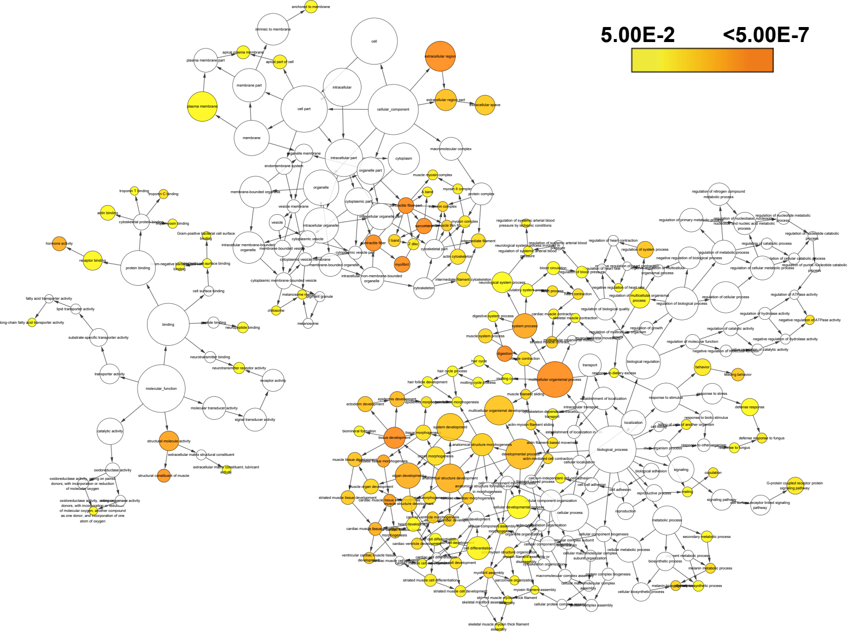

Figure S6. Directed acyclic graph of BiNGO analysis results of DEGs between low- and high-risk score.
